# Gut Microbiota Regulation of AHR Signaling in Liver Disease

**DOI:** 10.3390/biom12091244

**Published:** 2022-09-06

**Authors:** Baohong Wang, Ziyuan Zhou, Lanjuan Li

**Affiliations:** 1 State Key Laboratory for Diagnosis and Treatment of Infectious Diseases, National Clinical Research Center for Infectious Diseases, Collaborative Innovation Center for Diagnosis and Treatment of Infectious Diseases, The First Affiliated Hospital, Zhejiang University School of Medicine, Hangzhou 310003, China; 2 Research Units of Infectious disease and Microecology, Chinese Academy of Medical Sciences, Hangzhou 310022, China; 3 Jinan Microecological Biomedicine Shangdong Laboratory, Jinan 250021, China

**Keywords:** liver disease, gut microbiota, aryl hydrocarbon receptor, tryptophan

## Abstract

Liver health plays a vital role in human health and disease. Emerging evidence has shown the importance of the aryl hydrocarbon receptor (AHR) in liver diseases such as alcoholic liver disease, fatty liver disease, and liver failure. As a ligand-activated transcription factor, AHR can be activated by endogenous ligands of microbial metabolites such as tryptophan (Trp), kynurenine (Kyn) or indole derivatives locally or distantly. However, the therapeutic effects of the gut microbiota-regulated AHR pathway remain to be clarified. In this review, we summarize recent progress and examine the role of AHR signaling as a target for gut microbiota intervention in liver diseases. The focus on AHR signaling will identify a promising target in the gut microbiota for better understanding and therapeutic opportunities in liver diseases.

## 1. Introduction

Liver disease remains a great challenge for human health. Due to the anatomical association of the gut and the liver, the gut microbiota and associated metabolites widely influence host physiology and pathology and show important value in multiple areas, including nutrition, metabolism and immunity. The human gut harbors the most diverse microbial communities, which are mutualistic factors via the coevolution between humans and the gut microbiota. The gut microbiota can regulate host physiology directly through cell-to-cell contacts and indirectly through microbial metabolites [1,2,3,4]. In addition to local effects triggered by direct contact, the gut microbiota can affect distant organs through the gut-liver axis. The complex enzyme system of the gut microbiota drives numerous distinct biochemical reactions, regulates the host immune system, and supplements host metabolism. Such abundant microbial metabolites endow the gut microbiota as an important endocrine organ. It is worth noting that the important microbial metabolite precursor dietary tryptophan (Trp) can be used by host cells and the intestinal microbiota and can be used to produce endogenous aryl hydrocarbon receptor (AHR) ligands [1]. These metabolic pathways establish a strong connection between the gut microbiota and AHR signaling in the gut-liver axis.

Recent studies have raised interest in the relationship between the AHR pathway and liver diseases, including alcoholic liver disease (ALD) [5], nonalcoholic fatty liver disease (NAFLD) [6], and liver failure [7,8]. AHR, which is a ligand-activated multifunctional transcription factor [9], is expressed in various tissues and cells, such as CD4^+^ T cells and macrophages, and participates in processes including the mucosal barrier [10], cell differentiation [11], cancer [12], immunity and inflammation [13,14,15,16]. The therapeutic potential of AHR, such as regulating inflammation and cytoprotection, has been identified by different studies [5,17,18]. Considering the strong link between the gut microbiota and AHR signaling, it is feasible to explore gut microbiota modulation of the AHR pathway as a potential therapeutic target for liver diseases.

## 2. The AHR Signaling Pathway

**Figure 1:**
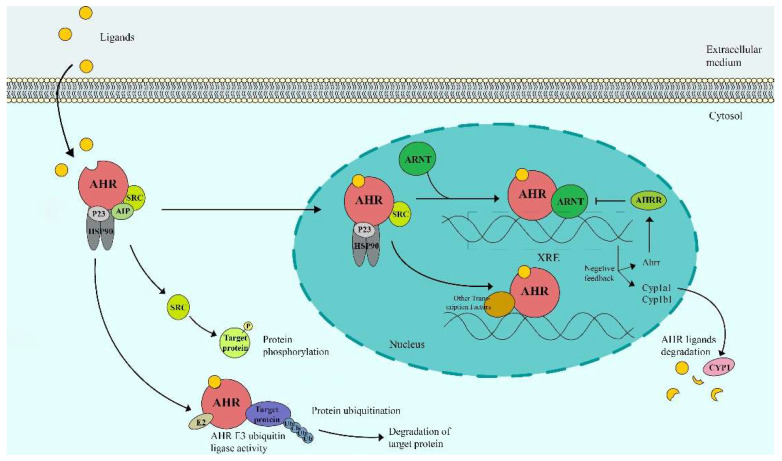
The cellular Aryl hydrocarbon receptor (AHR) signaling pathway. Once the different agonists bind to the AHR receptor, conformational changes in the complex appeared, leading to the activation of AHR, nuclear translocation, and a series of the downstream gene expression, which plays an important role in the inflammation-related diseases. Abbreviations: HSP90,90 kDa heat shock protein; AIP, AHR-interacting protein; ARNT, AHR nuclear translocator; XREs, xenobiotic response elements; AHRR, aryl hydrocarbon receptor repressor; CYP, Cytochrome P450.

AHR is a ligand-activated transcription factor in the Per-Arnt-Sim (PAS) superfamily that responds to various environmental signals, including dioxins and polycyclic aromatic hydrocarbons [19]. The cellular AHR signaling pathway is presented in Figure 1. In the inactive state, AHR resides in the cytoplasm in a protein complex consisting of the 90 kDa heat shock protein (HSP90), AHR-interacting protein (AIP), cochaperone p23 and the protein kinase c-Src, and is protected from degradation and translocation into the nucleus. In the active state, ligand binding changes the conformation of AHR, leading to protein complex dissociation and nuclear translocation. After entering the nucleus, AHR regulates the expression of many genes. Mostly, AHR binds to the AHR nuclear translocator (ARNT), regulating the expression of target genes containing AHR-responsive DNA elements (called xenobiotic response elements (XREs)) [18,20,21]. In addition, AHR can bind to other transcription factors, such as NF-κB, the retinoic acid receptor and the oestrogen receptor [22]. In addition to the genomic pathway, AHR also modulates biological processes in nongenomic patterns, including the protein kinase c-Src and calcium release. Moreover, AHR can act as an E3 ubiquitin ligase that participates in ubiquitylation and degradation driven by the proteasome [18,23]. Once the different agonists bind to the AHR receptor, conformational changes in the complex appeared, leading to a series of the downstream gene expressions. In general, the activated AHR signaling pathway could improve the inflammation-related diseases, such as ulcerative colitis [24], NASH, and skin inflammatory disease [25]. In the process, it could regulate the function of immune cells in a variety of pathways, including the AHR/glycolysis axis, the AHR/Nrf2/NQO1 pathway, and the AHR/CYP1A1 axis [26]. Typically, a rapid negative feedback loop limits AHR activation. The downstream cytochrome P450-dependent monooxygenases CYP1A1 and CYP1A2 can accelerate the degradation of the AHR ligand [14]. Thus, the cellular AHR signaling pathway can be activated by endogenous ligands of microbial metabolites, which might be a target for gut microbiota intervention for liver inflammation disease, which plays an important role in the inflammatory pathway in the AHR-microbiome-liver axis.

## 3. AHR Ligands

AHR was originally identified by its ability to respond to dioxin and other pollutants, and high-affinity ligands of AHR, such as dietary phytochemicals and microbial products, have been identified [27]. However, it is noteworthy that different AHR ligands or different concentrations of the same ligands can exert distinct biological characteristics. For example, an animal study showed that a normal concentration of FICZ (50 µg/kg) was rapidly metabolized by the negative feedback loop, while TCDD (15 µg/kg) or a high concentration of FICZ (10 mg/kg) was minimally metabolized and could induce the sustained activation of AHR. TCDD and FICZ, which are two high-affinity AHR ligands, have been reported to have opposite effects on T-cell differentiation, which is attributed to the concentration and the duration of AHR activation by high-affinity AHR ligands and affects T-cell differentiation [28].

In recent decades, the metabolites of Trp produced by the microbiota or host cells from the diet are novel AHR agonists that have been shown to be involved in various diseases, especially liver disease [29]. Trp can be metabolized by the microbiota into indole derivatives that are AHR agonists. However, host cells can metabolize Trp mainly by the kynurenine (Kyn) pathway, the metabolites of which are also AHR agonists [1]. Since cells and the microbiota use the same source, there may be competition between the two pathways. In other words, the mechanism by which the AHR signaling pathway is regulated by metabolites is complex, and the connection between these factors should be examined further.

## 4. The Metabolism of the Microbiota Regulates the AHR Pathway

The gut is the largest microbial habitat, harbouring more than 70% of the microbes in humans [2]. With long-term coevolution, the gut microbiota, which has large numbers and diversity, has developed a complex enzyme system to supplement the human host. This microbial metabolism is important for multiple physiological functions. Microbial regulation of AHR signaling has long been observed. Trp is the source of endogenous AHR ligands [1,30,31]. As one of the most well-described microbial metabolism pathways, Trp metabolism is closely related to AHR signaling and consists of one pathway that involves the transformation of Trp by the gut microbiota, the indole derivative pathway, and two pathways involving the transformation of Trp by the host cells: the Kyn pathway and the serotonin (5-hydroxytryptamine [5-HT]) production pathway, which the gut microbiota could act on [1].

### 4.1. Trp Metabolism by the Microbiota through the Indole Derivative Pathway

**Figure 2:**
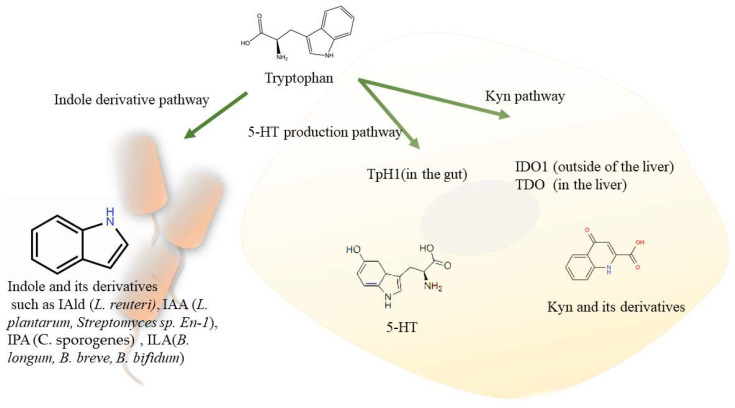
Microbial regulation of host-metabolized tryptophan pathways. Tryptophan could be metabolized to indole and its derivatives via indole derivative pathways in bacteria (*L. reuteri*, *L. plantarum*, and *C. sporogenes*), such as IAld, IAA, and IPA, respectively. Meanwhile, tryptophan could be metabolized to TpH1 in host cells via 5-HT production pathways as well as IDO1 (outside of the liver) and TDO (in the liver) via Kyn pathways. Abbreviations: IAld, indole-3-aldehyde; IAA, indole acetic acid; IPA, indole propionic acid; TpH1, Tryptophan hydroxylase I; 5-HT, 5-hydroxytryptamine; IDO1, indoleamine 2,3-dioxygenase 1; TDO, Trp-2,3-dioxygenase; Kyn, kynurenine.

Some dietary Trp is directly used and catabolized by commensal species into indole and its derivatives, such as indole-3-aldehyde (IAld), indole-3-acid-acetic (IAA), and indole-propionic acid (IPA) (Figure 2) [1]. In germ-free or dysbiotic mice, AHR agonists in the intestine are significantly deficient [32]. Interestingly, the metabolites of tryptophan had the cytostatic properties in models of breast cancer via activating AHR [33,34]. Thus, the microbial transformation of Trp is an important source of endogenous AHR ligands [1,33,34,35].

To date, a few Trp-metabolizing microbial species that can activate AHR have been identified (Table 1), but more have yet to be explored. For example, the well-studied probiotic *Lactobacillus* spp. contains many species that can produce AHR ligands. Targeted metabolomic analysis revealed that Trp catabolism by *L. reuteri* produces the indole derivative IAld through the indole pyruvate pathway via the aromatic amino acid aminotransferase [17]. Reduced production of AHR ligands was associated with reductions in three *Lactobacillus* strains in inflammatory bowel disease, and colitis was attenuated after mice were inoculated with three *Lactobacillus* strains that could metabolize Trp to indole derivatives, such as IAA [32]. A computational analysis revealed that *Peptostreptococcus russellii* could use mucin to produce the Trp metabolite IAA, thus promoting intestinal epithelial barrier function and mitigating inflammatory responses [36]. Laursen et al. demonstrated that *Bifidobacterium* species were able to convert Trp into IA and activate AHR in vitro [37]. Recently, *Bacteroides* spp. were also found to be involved in Trp catabolism. One study showed that pectin supplementation increased *Bacteroides* and indole derivatives, which enhanced colon and liver AHR signaling in mice [5]. A further study verified the ability of *Bacteroides thetaiotaomicron* to activate AHR signaling and ameliorate inflammation [38].

Some microbial Trp-metabolizing pathways have been identified. The direct decarboxylation of Trp in bacteria such as *Clostridium sporogenes* is a source of the AHR ligand tryptamine [45]. Through the indole-3-acetamide pathway, IAA is converted from Trp via the enzyme tryptophan 2-monooxygenase in *Fusarium* species [47]. In addition, several commensal species produce IAA and IPA through oxidative and reductive pathways [1]. *E. coli* and *Lactobacilli* convert Trp into indole via tryptophanase [35]. Discovering more pathways can better elucidate microbial AHR signaling regulation and contribute much to uncovering unknown Trp-metabolizing species. In addition, further studies need to enlarge the exploration on both the identity and concentration of the bacterial metabolites of the AHR ligands in serum or in the relevant tissues.

### 4.2. Microbial Regulation of Host-Metabolized Trp Pathways

More than 95% of dietary Trp is converted through the Kyn pathway by the rate-limiting enzyme IDO1 (Indoleamine 2,3-Dioxygenase 1) outside of the liver and by Trp-2,3-dioxygenase (TDO) in the liver. The Kyn pathway is important for the biosynthesis of nicotinic acid and nicotinamide adenine dinucleotide (NAD+) [48,49]. Kyn and its derivatives serve as AHR agonists and key regulators of inflammation, and the Kyn pathway is important in the immune system. Inflammatory factors, such as interferon-γ (IFN-γ), lipopolysaccharide (LPS), and prostaglandin E2 (PGE2) [50,51,52], stimulate IDO expression, enhancing Kyn production, which is characterized by decreased Trp and increased Kyn in the circulation [49,50].

Many studies have demonstrated the indirect regulatory role of the gut microbiota in the Kyn pathway. Germ-free (GF) animals and antibiotic-induced microbiota depletion in mice after weaning showed reduced Kyn pathway metabolism [53], suggesting a critical role for the gut microbiota in Kyn metabolism. In contrast, other probiotic intervention studies revealed the negative effect of microbial factors on Kyn metabolism. For example, indole-producing *Lactobacillus* species such as *Lactobacillus johnsonii* could inhibit IDO expression, lowering Kyn concentration in rats [54], and supplementation with the probiotic *Lactobacillus plantarum* 299v decreased Kyn concentrations in humans [55].

High levels of Kyn in the plasma and faeces are associated with a deleterious metabolic profile in the context of obesity because the increase in IDO activity shifts Trp metabolism from the generation of indole derivatives towards Kyn production. Interestingly, the deletion or inhibition of IDO improved obesity by shifting Trp metabolism by the gut microbiome towards the generation of indole derivatives that were related to cytokines [56]. In summary, gut microbes modulate the body’s pathophysiological process (e.g., inflammation) by competing with host cells for Trp metabolism.

The serotonin pathway is a metabolic pathway in which Trp is converted to 5-HT by the enzyme Trp hydroxylase 1 (TpH1) in the gut and particularly in enterochromaffin cells (ECs) [1]. The gut microbiota was shown to be associated with intestinal 5-HT production. The short-chain fatty acids (SCFAs) and secondary bile acids produced by the microbiota stimulate TpH1 expression, impairing 5-HT production in the colon and reducing 5-HT concentrations in the blood of GF mice [57]. Moreover, Rosser et al. found that a new AHR ligand, the serotonin-derived metabolite 5-hydroxyindole-3-acetic acid (5-HIAA), was increased by butyrate supplementation [58]. As shown in Figure 2, the major of tryptophan could be metabolized to TpH1 in the host cell in the gut via the 5-HT production pathway as well as IDO1 (outside of the liver) and TDO (in the liver) via the Kyn pathway. Tryptophan metabolic pathways could be regulated by the bacteria and metabolized to indole and its derivatives such as IAld, IAA, and IPA via indole derivative pathways in bacteria.

The diversity of AHR ligands and the complex metabolic network of Trp in both the microbiota and the host indicate the important role of AHR in various biological activities.

## 5. AHR in the Gut-Liver Axis: A Microbial Ecological Therapeutic Target for Liver Diseases

The liver is the primary organ that processes gut-derived microbial signals such as bacterial components or metabolites. The physiological interaction between the liver and the gut is a novel therapeutic target for many diseases and has been examined in many studies [59,60,61]. As discussed previously, the gut microbiota is critical in AHR signaling regulation. Gut microbiota regulation of AHR has attracted much attention as a key regulator of inflammation, barrier function and cell regeneration in multiple organs, including the liver [62,63], gut [16,64] and brain [18,65].

Endogenous AHR ligands produced by the gut microbiota can affect the gut and liver. Here, we list the most reported AHR ligands in different origins and their roles in liver diseases (Table 2). AHR signaling is known to play a critical role in intestinal immunity by maintaining epithelial barrier integrity and alleviating excessive inflammation [66,67]. One possible mechanism is the AHR-interleukin-22 (IL-22) pathway. IL-22 is a critical regulator of epithelial homeostasis, including the regulation of epithelial renewal and permeability and the production of mucus and antimicrobial proteins (AMPs) [68]. The presence of innate lymphoid cells (ILCs), which specialize in the secretion of large amounts of IL-22 in the intestinal lamina propria, has been shown to be AHR-dependent [69,70]. In addition, Zelante T et al. found that indole derivatives, which are AHR ligands produced by several *Lactobacillus* species, can induce the expression of IL-22, thus improving mucosal immunoreactivity against infection and inflammation [17]. Prevailing evidence has highlighted impaired intestinal barrier integrity and gut permeability as pathogenic factors in many liver diseases, which in turn has led to extensive attention on AHR signaling.

**Figure 3:**
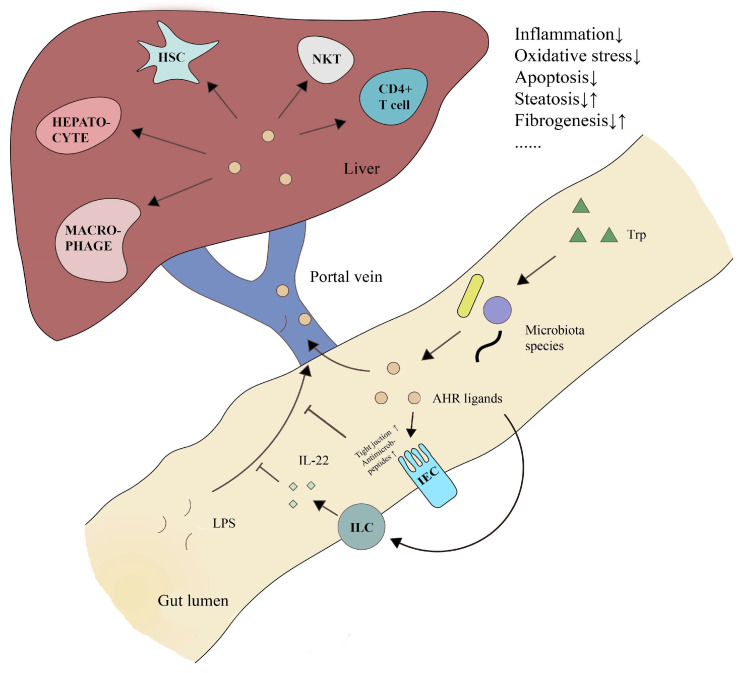
Gut microbiota-regulation of AHR signaling in liver physiology and pathology. Specific microbiota species (e.g., *Lactobacillus* spp.) can use and degrade dietary Trp, producing endogenous AHR ligands (mainly indole derivatives). On the one hand, AHR signaling can improve tight junction and antimicrobial peptide expression in IECs and the IL-22 production in ILCs, thus enhancing epithelial integrity and intestinal homeostasis. Under pathological conditions, AHR ligands are beneficial for improving gut permeability and blocking endotoxin translocation. On the other hand, previous studies have revealed that AHR signaling plays an important role in multiple cell types, especially immune cells. Gut-derived AHR ligands can regulate target cells in the liver through portal vein transmission. Generally, AHR signaling is characterized as an anti-inflammatory factor that protects against pathogenic inflammation and oxidative stress, exhibiting therapeutic value in multiple liver diseases. However, under different conditions, AHR signaling can induce fibrogenesis and steatosis. Thus, the regulation of AHR signaling by altering the gut microbiota could be a microbial ecological therapeutic method for liver diseases. Abbreviations: Trp, tryptophan; AHR, aryl hydrocarbon receptor; IEC, intestinal epithelial cell; LPS, Lipopolysaccharide; ILC, innate lymphoid cell; HSC, hepatic stellate cell; IL-22, interleukin-22; NKT, natural killer T cell.

On the other hand, AHR signaling exerts a direct effect on the liver. Multiple AHR-targeted cells, including hepatocytes [74,77], hepatic stellate cells (HSCs) [62], macrophages [77,84], natural killer T (NKT) cells [85] and CD4^+^ T cells [11,40], are critical or potential players in different liver diseases. For example, treatment with the AHR ligand indole inhibited LPS-induced inflammation in primary liver cell lines and alleviated hepatic inflammation in LPS-challenged mice [83]. Additionally, S Krishnan et al. showed that IAA could attenuate inflammatory indicators in macrophages and cytokine-mediated lipogenesis in hepatocytes in vitro [77]. The indole derivatives exhibited great value in controlling inflammation and showed direct evidence of AHR activation. Prebiotic and probiotic supplementation enriched AHR ligands and upregulated liver AHR signaling [5]. Thus, we hypothesized that there was a distant effect of gut microbiota-regulated AHR signaling on the liver through the portal vein system (Figure 3).

**Figure 4:**
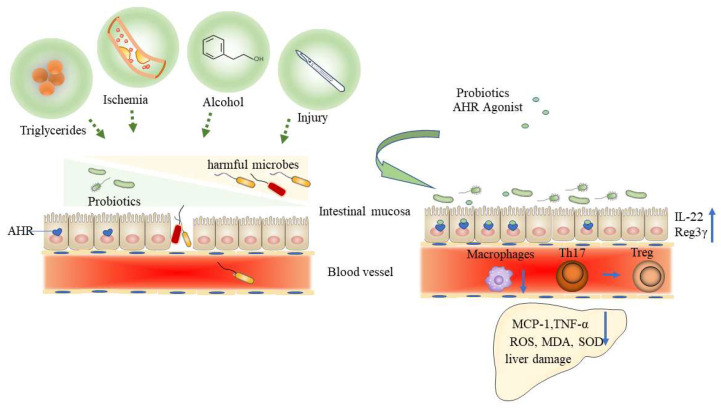
Gut microbiota-regulation of AHR signaling in the liver diseases. Triglycerides as in NAFLD, alcohol as in ALD, ischemia, surgical injury and other factors lead to the dysregulations of the microbiome and gut-derived metabolites, which can against the activation of the AHR, increase the intestinal permeability and incur the bacteria translocation into the blood. By providing probiotics or AHR agonists, the AHR signaling pathway was activated to reduce the intestinal permeability, decrease the immune macrophages, inhibit the transformation of Th 17 cells into Treg cells, and finally enhance the intestinal expression of IL-22 and Reg3γ, which ultimately alleviate the liver damage via down-regulating the inflammatory factors such as MCP-1, TNF-α, ROS, MDA and SOD. Abbreviations: AHR, aryl hydrocarbon receptor; Treg, regulatory T cells; REG3γ, regenerating islet-derived protein; MCP-1, monocyte chemoattractant protein-1; TNF-α, tumor necrosis factor-α; ROS, reactive oxygen species; MDA, malondialdehyde; SOD, superoxide dismutase.

A growing number of studies on liver diseases have focused on microbial ecology interventions to regulate AHR signaling, including antibiotics, prebiotics/probiotics and faecal microbiota transplantation (FMT) [5,8,32,86]. These findings shed light on the novel therapeutic approach to liver disease (Figure 4).

### 5.1. Alcoholic Liver Disease

ALD is a wide spectrum of diseases ranging from the asymptomatic to the development of hepatitis, fibrosis or cirrhosis. As a heavy burden on public health, ALD is a leading cause of liver-related morbidity and mortality worldwide [87,88]. Currently, the full mechanisms of ALD pathogenesis remain unclear.

The gut microbiota is believed to participate in the pathogenesis of ALD, and AHR may be a critical mediator. Early studies on ALD identified increased levels of endotoxin in peripheral serum samples and portal circulation, as well as gut dysbiosis, indicating disrupted intestinal epithelial barrier function in ALD [88,89]. In addition, chronic alcohol consumption was associated with downregulated intestinal expression of the antimicrobial peptides Reg3β and Reg3γ, intestinal dysbiosis and bacterial translocation [90]. Impaired intestinal barrier integrity exacerbates ethanol-induced liver injury. To our knowledge, AHR signaling is essential for intestinal stem cell homeostasis and IL-22 production, thus maintaining intestinal barrier integrity [10,13]. Reduced AHR signaling may be responsible for the impaired intestinal barrier function.

Consistent with our hypothesis, the supplement of probiotic *Lactobacillus rhamnosus GG* (LGG) was beneficial to experimental ALD by reinforcing the intestinal barrier function, which was likely mediated by bacterial AhR ligand-enriched LDNPs that increased the expression of Reg3 and Nrf2 [86]. Hendrikx et al. observed decreased intestinal levels of IAA and decreased activation of AHR in ALD mice, which downregulated intestinal IL-22 and Reg3γ expression, while supplementation with IAA improved liver injury by reversing the changes in IL-22 and Reg3γ expression [91]. To further elucidate the role of AHR in ALD, M. Qian et al. generated mice with intestinal epithelial cell-specific AHR deficiency (AHRΔIEC mice), which were administered ethanol. Unsurprisingly, AHRΔIEC mice exhibited severe liver injury after ethanol administration. Researchers then collected faecal samples from ALD patients and found that the mRNA and protein expression of intestinal AHR was significantly decreased [73]. These findings suggest an important role of the AHR signaling pathway in ALD.

Other researchers performed gut microbiota interventions and revealed the therapeutic effect of AHR signaling in ALD. For example, Ferrere G et al. performed FMT from alcohol-fed mice who were resistant to ALD to normal mice. Interestingly, alcohol resistance was successfully replicated in the alcohol-fed mice that received FMT [92]. Pectin, which is a galacturonic acid-rich polysaccharide, can strengthen the mucus layer, enhance epithelial integrity, activate or inhibit dendritic cell and macrophage responses through pattern recognition receptors, and stimulate the diversity and abundance of beneficial microbial communities [93], resulting in resistance to ALD. Gut microbiota analysis revealed similar characteristics, including the reversed proportion of *Bacteroides* [92]. Following this finding, Wrzosek L et al. performed further experiments with pectin treatment in human-associated mice and identified AHR as the target pathway. A pectin-rich diet increased *Bacteroides* abundance, which accounted for the increase in indole derivatives [5]. Another Trp-degrading species, *Lactobacillus rhamnosus GG,* which showed a positive effect on experimental ALD [94,95], was shown to improve ALD through intestinal AhR-IL-22-related signaling pathways, reducing the level of bacterial translocation and LPS release [86].

### 5.2. Nonalcoholic Fatty Liver Disease

Currently, NAFLD is one of the leading causes of chronic liver disease globally due to the rapidly increasing levels of obesity and metabolic syndrome. Developing from nonalcoholic fatty liver (NAFL) to nonalcoholic steatohepatitis (NASH), NAFLD has become a rising threat that leads to cirrhosis and liver cancer [96,97].

It is quite clear that the gut microbiota is involved in NAFLD progression, but the role of AHR is still controversial. Previous findings mostly showed that AHR signaling was a pathogenic factor in steatosis development. Lee et al. performed a deep examination of AHR with constitutively activated AHR transgenic animals. Constitutive activation of AHR induced spontaneous hepatic steatosis, which was dependent on the increased expression of fatty acid translocase and fatty acid transport proteins [98]. A similar effect was observed in a series of studies that [71,72,99] highly suggested a preventive target for NAFLD.

Recent animal studies have indicated a different conclusion. As we described previously, indole derivatives have beneficial effects on intestinal immunity, and epithelial homeostasis is a key factor in liver physiology. Zhao et al. demonstrated that the administration of IPA not only modulated intestinal homeostasis, decreased LPS levels and inhibited liver inflammation but also downregulated fibrogenic and collagen genes and alleviated HFD-induced NASH phenotypes [80]. In addition, the oral intake of IAA relieved NAFLD and decreased the macrophage ratio, which mainly resulted in the production of anti-inflammatory factors in vivo and in vitro [79]. Furthermore, Ji et al. demonstrated the protective effect of IAA against HFD-induced oxidative stress, such as reactive oxygen species (ROS) and malonaldehyde (MDA) levels, along with superoxide dismutase (SOD) activity. The inflammatory response of the liver in HFD mice was significantly ameliorated, which was characterized by reduced F4/80-positive macrophage infiltration and monocyte chemoattractant protein-1 (MCP-1) and tumour necrosis factor-α (TNF-α) expression [78]. Xu et al. revealed similar outcomes as well [92]. These findings suggest that indole derivatives can be therapeutic targets in NAFLD, although direct evidence of AHR dependence is still lacking. Interestingly, a recent study by Shi, Z et al. showed that in saccharin/sucralose-induced NAFLD mice, the gut microbial community structure was altered, there was a significant reduction in *Akkermansia muciniphila* abundance, and colonic AHR signaling and AHR ligand concentrations were reduced. Metformin or fructo-oligosaccharide supplementation ameliorated NAFLD and restored *A. muciniphila* and AHR ligands [6], which may suggest the therapeutic value of AHR-related commensal species. Recently, in addition to the probiotics or fiber/prebiotics, the herbs received attention for the prebiotic role in the gut, such as ganoderma lucidum polysaccharides [100]. Additionally, *M. fragrans* extract improved the inflammation and lipid metabolism by activating the tryptophan metabolite mediated AHR in NAFLD [101].

The controversial role of AHR in NAFLD may be due to different ligand species and activation durations. Further examination of various ways by which AHR is activated may uncover a deeper connection between AHR and NAFLD.

### 5.3. Acute Liver Failure

Acute liver failure (ALF) is a multicausal clinical syndrome characterized by fulminic hepatocyte necrosis. The rapid pathologic progression limits the efficacy of regular internal medical treatment and typically leads to poor outcomes [102].

To date, direct evidence for gut microbiota intervention therapy in ALF is lacking, but several studies have raised interest in IL-22, the critical factor in the regulation of inflammation induced by AHR. Ashour T. H studied the therapeutic efficacy of IL-22 administration on liver injury in an ALF rat model induced by D-galactosamine (D-GalN)/LPS. Liver injury (ALF, AST, histopathological scores) and survival curves (survival time and rate) were significantly improved. In addition, hepatic levels of TNF-α, which is the major pathogenic factor in ALF, were decreased, indicating the anti-inflammatory role of IL-22 in ALF [103]. In addition, the protective effect of IL-22 was identified in acetaminophen-induced, ischaemia reperfusion-induced and concanavalin A-induced acute liver injury [104,105,106]. Early-stage administration of IL-22 may be beneficial for blocking TNF-α-induced liver inflammation, protecting hepatocytes from large-scale necrosis. One possible idea is the prevention of gut-derived LPS, which exacerbates ALF via toll-like receptor 4 (TLR4) [107].

Interestingly, another study indicated a direct connection between IL-22 and the gut microbiota in ALF. FMT from donor mice that were administered *Saccharomyces boulardii* by gavage significantly alleviated liver injury and ameliorated gut dysbiosis in D-GalN-induced ALF model mice. Further analysis revealed the increased expression of interleukin-10 (IL-10) and IL-22 and reduced expression of interleukin-17A (IL-17A), indicating a shift from T helper type 17 (Th17) cells towards regulatory T (Treg) cells [108]. Consistent with previous findings, AHR signaling could induce a similar shift in Th17/Treg cell differentiation, which was characterized by upregulation of the Treg cell-related anti-inflammatory cytokines IL-10 and IL-22 [11,18]. In addition, research has demonstrated a protective role of *Lactobacillus* in ALF animals, which may be related to the productions of AHR ligands by the microbiota [109,110,111]. Although direct verification of AHR signaling involvement was lacking in these studies, this consistency suggests a possible connection between gut microbiota-regulated AHR and IL-22 treatment in the ALF model.

Additionally, HSCs, which are responsible for fibrogenesis and are activated during liver injury as proinflammatory factors [112], exhibited AHR dependence. The expression of AHR in murine HSCs decreased rapidly with HSC activation, and AHR–/–HSCs exhibited increased spontaneous activation. AHR ligands prevent HSC activation [62]. Moreover, a study by Stewart et al. generated a novel mouse model with depleted stellate cells that was protected against ischaemia/reperfusion- and endotoxin-induced ALF, indicating the pathogenic role of HSCs in ALF [113]. Thus, we suggest that HSCs might be a therapeutic target in ALF and are regulated by AHR signaling.

Generally, AHR signaling is one of the critical points in the gut-liver axis, showing therapeutic potential in liver diseases. First, gut microbiota intervention can regulate local AHR signaling. The microbiota-regulated AHR-IL-22 pathway and antimicrobial peptide expression have been shown to be essential factors in intestinal homeostasis, exerting protective effects on epithelial integrity and preventing gut dysbiosis and endotoxin leakage [10,13,17]. Second, the transmission of gut-derived AHR ligands provides a much milder way to regulate hepatic AHR signaling. Existing methods of microbiota intervention showed the curative effects of many endogenous AHR ligands, such as alleviating alcohol-induced lipid accumulation and hepatocyte lesions in ALD [5,92] and ameliorating the endotoxin/drug-induced acute phase in ALF [83,108]. These ambiguous aspects of AHR signaling in liver fibrogenesis [82,114] and its influence on NAFLD [6,72,78] suggest a therapeutic or preventive target. These findings require further verification. In brief, these publications show that AHR is an effective and promising target for gut microbiota intervention for the treatment of liver diseases.

## 6. Summary

With the rapid progress in the understanding of the gut-liver axis, the gut microbiota exhibits increasing therapeutic value, and a number of novel targets in liver diseases have been identified [115]. However, gut microbiota-regulated AHR signaling pathways exhibit high complexity and are not well understood. The broad spectrum of targeted cells, especially immune cells, indicates robust therapeutic potential. Unlike many toxic xenobiotic ligands, endogenous microbial ligands are much milder and more feasible. However, different ligands and treatment durations lead to diverse physiological responses. Accurate examination of microbial AHR-related metabolism under various experimental conditions is required to clarify this concern. However, existing studies have mostly been performed on rodent models, and human AHR may differ in ligand selectivity and physiologic responses. Therefore, pilot studies on AHR-targeted gut microbiota intervention in patients are needed.

## Figures and Tables

**Table 1:** AHR-activating-microbiota species.

Species	Metabolites
*Lactobacillus*	
*L. reuteri* [17,32,39,40,41]	IAld (Indole-3-aldehyde) [17,39]
*L. murinus* [32]	-
*L. taiwanensis* [32]	-
*L. johnsonii* [17]	ILA (Indole-3-lactic acid) [17]
*L. plantarum* [42]	IAA (Indole acetic acid) [42]
*L. bulgaricus* [43]	-
*Bifidobacterium*	
*B. longum* [37]	ILA [37]
*B. breve* [37]	ILA [37]
*B. bifidum* [37]	ILA [37]
*Peptostreptococcus*	
*P. russellii* [36]	IA (Indole acrylic acid) [36]
*Streptomyces*	
*Streptomyces sp. En-1* [44]	IAA [44]
*Bacteroides*	
*B. thetaiotaomicron* [5,38]	-
*Clostridium*	
*C. sporogenes* [45]	Tryptamine [45]
	IPA (Indole propionic acid) [46]
*Ruminococcu*	
*R. gnavus* [45]	Tryptamine [45]
Unknown	
Unknown [33]	Indoxylsulfate [33]
Unknown [34]	IPA [34]

Note: “-” means that no metabolites detection was performed in the study.

**Table 2:** The role of AHR ligands in liver diseases.

Compound	Origin	Effect
TCDD (2,3,7,8-tetrachlorodibenzo-p-dioxin)	Exogenous	Worsens hepatic steatosis and increases liver collagen staining and serum transaminase levels in the HFD mice [71]
		Prevents HSC activation and expression of genes required for liver Fibrogenesis [62]
		Sensitizes mice to NASH by inhibiting SOD2 activity, increasing ROS production, and increasing lipid peroxidation [72]
FICZ (6-Formylindolo [3,2-b] carbazole)	Host metabolism	Alleviates alcohol-induced liver injury and improves intestinal anti-microbial peptide levels [5]
		Protects mice from ALD by activating intestinal AHR without affecting liver AHR function [73]
		Protects ConA-induced liver injury via promoting IL-22 production from innate lymphoid cells and suppressing IFN-γ expression from NK T cells [70]
ITE 2-(1′H-indole-3′-carbonyl)-thiazole	Host metabolism	Sensitizes hepatocytes to hyperacute acetaminophen-induced hepatotoxicity by cyp1a2 activation [74]
		Prevents HSC activation and expression of genes required for liver fibrogenesis [62]
Kyn (Kynurenine)	Host metabolism	Exacerbates acute liver injury induced by carbon tetrachloride [75]
KYNA (Kynurenic acid)	Host metabolism	Attenuates thioacetamide- induced liver injury via elevating IL-10 levels [76]
IAA	Microbiota metabolism	Attenuates inflammatory response and reduces the expression of fatty acid synthase and sterol regulatory element-binding protein-1c in HFD mice [77] Alleviates NAFLD in mice via attenuation the hepatic lipogenesis, oxidative and inflammatory stress [78,79]
IPA (Indole-3-propionic acid)	Microbiota metabolism	Inhibits endotoxin leakage to attenuate steatohepatitis [80]
		Reduces cell adhesion, cell migration and mRNA gene expression in human HSCs (LX-2) cells [81]
Tryptamin	Microbiota metabolism	Reduces fatty-acid- and LPS-stimulated production of pro-inflammatory cytokines in macrophages and inhibits the migration of cells toward a chemokine in HFD mice [77]
ICA (Indole-3-carboxaldehyde)	Microbiota metabolism	Restores gut mucosal integrity and protects against liver fibrosis in murine sclerosing cholangitis [82]
Indole	Dietary and microbiota metabolism	Dose-dependently reduces the LPS-induced up-regulation of proinflammatory mediators at both mRNA and protein levels partly via kupffer cells [83]
I3C (Indole-3-carbinol)	Dietary	Protects mice from ALD specifically by activating intestinal AHR without affecting liver AHR [73]

Abbreviations: AHR, aryl hydrocarbon receptor; HFD, high-fat diet; HSC, hepatic stellate cell; NASH, non-alcoholic steatohepatitis; ROS, reactive oxygen species; SOD2, superoxide dismutase 2; ALD, Alcoholic liver diseases; ConA, concanavalin A; IFN-γ, interferon-gamma; NK, natural killer; interleukin, IL; NAFLD, non-alcoholic fatty liver disease; LPS, Lipopolysaccharide.
